# *HMAP*, World Hepatitis Day and the bigger health systems picture

**DOI:** 10.1186/s41124-016-0013-9

**Published:** 2016-07-27

**Authors:** Jeffrey V. Lazarus, Kelly Safreed-Harmon, Mojca Maticic

**Affiliations:** 1 CHIP, Centre for Health and Infectious Disease Research and WHO Collaborating Centre on HIV and Viral Hepatitis, Rigshospitalet, University of Copenhagen, Copenhagen, Denmark; 2 ISGlobal, Hospital Clinic, University of Barcelona, Barcelona, Spain; 3 Clinic for Infectious Diseases and Febrile Illnesses, University Medical Centre, Ljubljana, Slovenia

## Abstract

World Hepatitis Day 2016 marks one year since *Hepatology, Medicine and Policy* (*HMAP*) began accepting submissions. There have been many major developments in the fight against hepatitis B virus (HBV) and hepatitis C virus (HCV) infections during this time, but there is no guarantee that the global community’s interest in these diseases will endure. Situating efforts to eliminate HBV and HCV within the movement to strengthen health systems is the key to ensuring that current momentum is sustained. Because of the very nature of viral hepatitis, an effective response requires the integration of many seemingly disparate components of health systems. Everyone working to end HBV and HCV epidemics thus has a vested interest in health systems strengthening. Furthermore, it is important for strategic purposes to look beyond what hepatitis stakeholders need from health systems, and to ask how the global response to HBV and HCV can help foster health systems innovations and put patient-centered care first. Addressing this question explicitly is central to ensuring that the long-term relevance of the viral hepatitis movement is recognized by diverse health and development actors.

## Editorial

*Hepatology, Medicine and Policy* (*HMAP*) came into being one year ago, on World Hepatitis Day 2015. Our call for submissions generated widespread interest, and we began publishing during the International Liver Congress (ILC) in April 2016. We are deeply indebted to the many experts who joined the editorial board of our fledgling journal, to the associate editors who oversee articles, to the authors who dared to submit to a new journal and to the staff at our publisher.

The past year has been eventful. The first World Hepatitis Summit brought together patients, healthcare professionals, government leaders, World Health Organization (WHO) officials and other stakeholders for three days of learning and strategizing in September 2015 [1]. The real-world efficacy and safety of powerful new direct-acting antiviral (DAA) treatments for hepatitis C virus (HCV) infection was confirmed in research presented at the ILC in April 2016 [2, 3]. WHO’s first-ever global health sector strategy on viral hepatitis was adopted in May 2016 [4]. The World Hepatitis Alliance and partners have recently introduced the “NOhep” campaign to end viral hepatitis [5], and the full elimination of one or more viral hepatitides is increasingly recognised as an attainable goal.

But what are we to make of the outpouring of interest in viral hepatitis and the much-increased visibility of this group of diseases? Is the world finally awakening to the magnitude of the problem? Or is this just a transitory phenomenon?

We raise the question because the medical and public health fields, in spite of aspiring to be the embodiment of rationality, are remarkably trend-driven in some regards. It is not unheard of for a disease or health issue to have its moment in the sun, suddenly enjoying an upsurge of interest among researchers, donors, government officials and the general public, then for the interest to dissipate. Just look at the current Zika virus epidemic, the 2014–2015 Ebola hemorrhagic fever outbreak, and influenza pandemics before that.

Many people in our circles have suggested that the years 2015 and 2016 mark a turning point in the global fight against HBV and HCV. Five years on, will we indeed look back at this as the time when a movement coalesced, and the stage was set for us to drive forcefully toward elimination of these two diseases? Or will it be remembered as a period when viral hepatitis briefly received the attention that was warranted, after which it was quickly forgotten?

When we think about how to capitalize on current opportunities, and how to ensure that momentum is sustained, we are struck by the importance of situating efforts to end HBV and HCV epidemics within the movement to strengthen health systems. The concept of “health systems strengthening” may seem like an abstraction to biomedical, technical and health service experts who are accustomed to thinking in terms of liver fibrosis stages, seroprevalence data and quantities of clean needles and syringes distributed to people who inject drugs. But we urge people to step back from their diverse areas of hepatitis-related expertise and take stock of how this work fits into health systems strengthening.

In making this proposal, we are thinking specifically of the conceptualization of health systems strengthening as “permanently making the system function better, not just filling gaps or supporting the system to produce better short-term outcomes” [6]. The latter approach is often motivated by health crises, for example a dramatic increase in the prevalence of a particular disease or a surge in demand for health services in the wake of a natural disaster. All too often it results in governments and international partners working in fragmented, episodic and inefficient ways to address individual public health challenges rather than building up the capacity of the health system to address a large constellation of current and future challenges in an integrated manner.

The Ebola epidemic dramatically exposed longstanding health system weaknesses in Guinea, Liberia and Sierra Leone, including financial, human resource and service delivery weaknesses [7, 8]. What many people may not realize is that less headline-friendly health system failures are occurring in countries at all income levels on an ongoing basis. These failures take myriad forms. Consider, for example, that almost one-quarter of HIV-positive people in the United Kingdom do not know their HIV status [9], and that more than half of the estimated 11 million undocumented immigrants residing in the United States lack any form of health insurance [10].

So what does the drive to eliminate HBV and HCV infections have to do with health systems strengthening? Well, everything. Because of the very nature of these diseases, an effective response requires the integration of many seemingly disparate components of health systems. In other words, those of us working to end HBV and HCV epidemics vitally need health systems strengthening efforts to be successful if we are to achieve our goals.

Furthermore, it is important for strategic purposes to look beyond what we need from health systems. How can the global response to viral hepatitis help to *foster* health systems innovations and put patient-centered care first? Addressing this question explicitly is the key to ensuring that the long-term relevance of the fight against viral hepatitis is recognized by diverse health and development actors.

Barriers to achieving full hepatitis B vaccine coverage among infants in some countries, for example, need to be considered from the standpoint of how well national health systems are addressing infant and childhood vaccination overall. Another issue that cannot be addressed successfully in isolation is viral hepatitis prevention among people who inject drugs. Meeting the health-related needs of this population requires cross-disciplinary collaboration among experts on multiple health issues, including addiction, other mental health issues, and various infectious and noncommunicable diseases. How might a health system’s full array of resources, including service delivery, financing and health information systems, be best configured to optimize health outcomes in light of these considerations?

Those of us working primarily on viral hepatitis-related issues do not have any easy answers—but we have a responsibility to explore how health system-related factors might enhance or undermine the effectiveness of the interventions that we wish to see brought to scale. We also can articulate the challenges of our work to the larger health systems community in ways that encourage beneficial changes. We might, for example, choose to press the question of whether the widespread convention of dichotomizing diseases as either infectious or noncommunicable has outlived its usefulness. Hepatitis B and hepatitis C are infectious diseases that give rise to liver cancer, cirrhosis and liver failure. In some settings, coinfection with multiple hepatitis viruses, as well as with HIV and other infectious agents, is not uncommon. Effective disease surveillance systems need to integrate data that are reflective of all of these considerations.

So this World Hepatitis Day, as we set our sights on the WHO goal of eliminating HBV and HCV as a major public health threat by 2030 [11], let’s think about how the response to viral hepatitis can become more holistic and how it can be contextualized for every health system, large and small, from the national to the local level. Enacting this vision will extend the benefits of our accomplishments to hundreds of millions more people worldwide.
